# NOVAGene: a nonlinear approach to improve biological resolution in weighted gene co-expression networks for low-variability datasets

**DOI:** 10.3389/fbinf.2026.1813626

**Published:** 2026-05-12

**Authors:** Junelle Rey C. Bacong, Jose Jr B. Nevado, Marissa M. Alejandria, Maria Sonia S. Salamat, Joseph Adrian L. Buensalido

**Affiliations:** 1 College of Medicine, University of the Philippines Manila, Manila, Philippines; 2 Institute of Human Genetics, National Institutes of Health - University of the Philippines Manila, Manila, Philippines; 3 Section of Infectious Diseases, Department of Medicine, Philippine General Hospital, Manila, Philippines

**Keywords:** gene co-expression networks, distance correlation, sepsis, transcriptomics, network medicine, bioinformatics

## Abstract

Weighted Gene Co-expression Network Analysis (WGCNA) is widely used for identifying co-expression modules in transcriptomic studies due to its computational efficiency. However, its reliance on linear models may limit sensitivity to complex, nonlinear gene interactions, particularly in datasets with low variability like sepsis and septic shock, where shared pathogenesis results in subtle transcriptional differences. To overcome these limitations, we introduce a novel approach called Nonlinear Overlapping Variability Analysis of Gene Networks or NOVAGene which directly contrasts the individual nonlinear gene co-expression networks between conditions, effectively mitigating the impact of sample heterogeneity. Using a local sepsis dataset, we evaluated WGCNA’s ability to capture co-expression modules across varying biological variability. While effective in highly variable datasets, such as those comparing diseased and healthy individuals, WGCNA struggled to differentiate the transcriptome from patients who progressed to septic shock from those who did not due to its reliance on linear approximations and sample pooling. In contrast, NOVAGene identifies biologically meaningful modules across both low and high variable datasets. It effectively distinguishes patients who will develop septic shock from those who will not, revealing shared core immune processes but distinct regulatory dynamics that differentiate these two clinical outcomes. Our enhancement to gene co-expression analysis using NOVAGene offers a more refined approach to sepsis prognostication, providing insights into the subset of biological processes that are innately dysregulated in patients who are susceptible to septic shock. NOVAGene may facilitate the detection of subtle variations in key biomarkers that could improve the prediction of disease outcomes. Despite its higher computational demands and potential statistical limitations, NOVAGene effectively captures nonlinear transcriptional patterns, enhancing the resolution of disease network analysis in biologically similar datasets with minimal transcriptional variation.

## 1 Introduction

Network analysis has become a standard approach for identifying association patterns among genes within a biological system (Gosak et al., 2022; Charitou et al., 2016). A network is built with nodes representing genes and edges indicating gene interactions, such as co-expression relationships. By identifying clusters of highly interconnected genes, referred to as modules, network analysis facilitates the discovery of functionally related gene groups and their underlying regulatory mechanisms.

One widely used network-based bioinformatics tool is Weighted Gene Co-expression Network Analysis (WGCNA). This method generates a pairwise linear correlation matrix from gene expression data obtained through microarray or RNA-seq experiments. It identifies gene modules based on co-expression patterns across samples and associates them to known biological pathways and clinical traits, offering insights into gene regulation and functional relationships within the dataset (Langfelder and Horvath, 2012; 2008). However, gene co-expression patterns are often nonlinear. For instance, transcription factors and other gene regulators can induce complex, nonlinear effects on their target genes (Kontio et al., 2020).

Majority of methods utilizing co-expression networks, including WGCNA, assume only linear relationships between genes due to its simplicity (Kontio et al., 2020; Wang et al., 2016). While linear approximations of gene co-expression patterns are often adequate for datasets with high variability, such as those comparing healthy controls to diseased samples, they may fall short when analyzing datasets with low variability, particularly in diseases sharing similar pathogenesis.

Sepsis and septic shock represent a disease continuum of the same pathophysiology. Defined by the Third International Consensus Definitions for Sepsis and Septic Shock (SEPSIS-3), sepsis is defined as life-threatening organ dysfunction caused by a dysregulated host response to infection and may progress to organ failure in severe cases, while septic shock is a more severe subset of sepsis characterized by profound circulatory and metabolic abnormalities associated with a higher risk of mortality (Singer et al., 2016). Since both conditions arise from the same underlying pathophysiology, they constitute a low-variability dataset, making traditional linear co-expression models less effective. The complexity of gene co-expression patterns in sepsis and septic shock may require more sophisticated models to capture the nuanced relationships accurately.

Recently, newer methods tried to incorporate nonlinear co-expression patterns in the standard pipeline of WGCNA after recognizing its limitations (Hou et al., 2022; Zhang and Wong, 2022). For example, Hou et al. (2022) showed that distance correlation is better at revealing complex biological relationships between gene profile compared to other correlation metrics in WGCNA such as Pearson, Spearman and Maximal Information Coefficient (MIC).

In this study, we present a novel approach called NOVAGene or Nonlinear Overlapping Variability Analysis of Gene Networks. NOVAGene integrates distance correlation with WGCNA to identify functional modules in low-variability gene expression datasets, such as those from sepsis and septic shock. Using a local sepsis dataset, we compared (1) healthy vs. diseased states, representing high variability, and (2) sepsis vs. septic shock, representing low variability. While NOVAGene successfully identified known pathways in the initial comparison, it also uncovered distinct biological processes that differentiate diseased patients prone to septic shock from those who are not. By capturing subtle regulatory differences, NOVAGene enhances functional module detection, offering deeper insights into gene regulation, biomarkers, and therapeutic targets for early intervention.

## 2 Methods

### 2.1 Patient recruitment and inclusion/exclusion criteria

This study used a local sepsis dataset from a previous prospective study (2015–2018) that recruited 340 patients (18–60 years) from two tertiary hospitals in Metro Manila, Philippines. The participants provided informed consent and were initially categorized into diseased group and healthy controls based on the systemic inflammatory response syndrome (SIRS) criteria (Marik and Taeb, 2017). The diseased group consisted of hemodynamically stable patients with confirmed infection and SIRS, but without respiratory distress or refractory hypotension. In contrast, healthy controls included individuals who have been free of infection-related symptoms for ≥3 months or do not meet the SIRS criteria. Patients with malignancies, chronic lung/liver disease, Stage V CKD, and chronic symptomatic infections were excluded in the study. Clinical outcomes were monitored for 28 days, with patients classified as either non-shock or shock based on the SEPSIS-3 definition (Singer et al., 2016).

### 2.2 Whole-genome expression profiling of collected samples

Human blood samples (3 mL) were collected in EDTA (ethylenediaminetetraacetic acid) tubes, processed within 2 hours, and PBMCs (peripheral blood mononuclear cells) were isolated and stored at −80 °C. RNA was extracted using Qiagen kits, and quality was assessed via NanoDrop and TapeStation. Whole-genome expression profiling was performed using Illumina HiScanSQ and Affymetrix GeneChip platforms, following standard protocols for RNA processing and hybridization. The intensity data were analyzed using Genome Studio Software and GeneChip Command Console.

### 2.3 Comparison groups based on affliction and clinical outcomes

We analyzed 119 human samples from the 340 recruited patients, of whom N=80 were classified as diseased and N=39 as healthy. Within the diseased group, patients were further stratified based on their clinical outcomes after 28 days into non-shock (N=61) and shock (N=19) groups. The non-shock group included patients who did not develop hypotension, whereas the shock group comprised those who had refractory hypotension (Singer et al., 2016). This stratification allowed for two primary comparisons: (1) the affliction-based comparison for diseased vs. healthy groups, and (2) the outcome-based comparison for shock vs. non-shock groups.

### 2.4 Different approaches to gene co-expression analysis

#### 2.4.1 Standard WGCNA pipeline

We performed weighted gene co-expression network analysis (WGCNA, RRID: SCR_003302) to both comparison groups using the WGCNA R package (Langfelder and Horvath, 2008). Pearson’s correlation (cor function) was used to construct a scale-free gene network, with a soft-thresholding power (β) chosen to achieve ${\text{R}}^{2}=0.9$ . The adjacency matrix was transformed into a topological overlap matrix (TOM) to capture both direct and indirect gene connections, enhancing the biological relevance of network connectivity. Gene modules (minimum size = 30) were identified using hierarchical clustering and assigned unique colors. To explore module-trait relationships, we calculated the module eigengene (ME) for each module–the first principal component summarizing gene expression within a module–and correlated it with clinical traits such as comparison group membership, mortality, and hospitalization.

#### 2.4.2 Distance correlation-based WGCNA

We explored the use of distance correlation instead of Pearson’s R to construct a scale-free gene network as prescribed by Hou et al. (2022). The distance correlation is a measure of joint independence of two random variables $X\in R^{p}$ , $Y\in R^{q}$ . The correlation is based on the non-negative empirical distance covariance $V_{n}\left(X,Y\right)$ defined by Székely et al. (2007) as

$$V_{n}^{2}\left(X,Y\right)=\frac{1}{n^{2}}\overset{n}{\underset{k,l=1}{\sum }}A_{kl}B_{kl},$$ (1)

where $A_{kl}=a_{kl}-{\bar{a}}_{k.}-{\bar{a}}_{.l}+{\bar{a}}_{.}$ and $B_{kl}=b_{kl}-{\bar{b}}_{k.}-{\bar{b}}_{.l}+{\bar{b}}_{.}$ such that $a_{kl}={\left\|X_{k}-X_{l}\right\|}_{p}$ and $b_{kl}={\left\|Y_{k}-Y_{l}\right\|}_{q}$ for k,l=1,2,…,n , where ${\left\|\cdot \right\|}_{\omega }$ denotes the Euclidean norm in $R^{\omega }$ . The normalized expression of distance correlation (Equation 1) is given by the correlation metric $R_{n}$ of the following form:

$$R_{n}\left(X,Y\right)={\left[\frac{V_{n}^{2}\left(X,Y\right)}{\sqrt{V_{n}^{2}\left(X\right)V_{n}^{2}\left(Y\right)}}\right]}^{1/2}$$ (2)

The value of $R_{n}\left(X,Y\right)\in \left[0,1\right]$ , where $R_{n}\left(X,Y\right)=0$ only if X and Y are completely independent in any arbitrary dimensions. We used the dcor package in Python to compute the normalized pairwise distance correlations among genes from Equation 2, thereby constructing a nonlinear co-expression network for the same comparison groups. This network was processed similarly to standard WGCNA pipeline, replacing the adjacency matrix with a nonlinear version before deriving the topological overlap matrix. Hierarchical clustering was also applied to detect nonlinearly coexpressed gene modules.

#### 2.4.3 Our NOVAGene pipeline

Unlike previous approaches that pool samples from datasets with varying heterogeneity, NOVAGene constructs separate gene co-expression networks for each condition–healthy, diseased, non-shock, and shockâ€“to enable direct structural comparisons. We used distance correlation to capture nonlinear gene-gene relationships and enforced a scale-free network topology, similar to standard WGCNA, by applying a soft-thresholding power β to the distance correlation matrix, ensuring $R^{2}=0.9$ . Enforcing a scale-free topology emphasizes strong and consistent connections while significantly diminishing the weak or unreliable ones.

To further enhance the signal-to-noise ratio, we fitted a powerlaw distribution to the network’s node strengths, defining theoretical parameters α and ${\text{x}}_{\min}$ . Nodes with strengths below ${\text{x}}_{\min}$ were removed, leaving a pruned network that retained an empirical powerlaw distribution–a characteristic observed in various biological networks (Simovic and Michaletz, 2025). We consequently performed a Kolmogorov-Smirnov (KS) statistics and calculated the likelihood ratio as goodness-of-fit tests to ensure that our local sepsis dataset indeed follows the correct heavy-tailed distribution. Similar to previous methods, the filtered network was also transformed into a topological overlap matrix. Subsequently, hierarchical clustering of the TOM-based dissimilarity matrix identified gene modules (minimum size: 30), each assigned a distinct color.

From the nonlinear gene co-expression networks constructed for each condition, we assessed the relationships and structural shifts between corresponding modules across comparison groups. Unlike existing module comparison methods–such as Z-summary in WGCNA, graphlet correlation distance, or the Jaccard index–which assess similarity based on module preservation, local topology, or membership overlap, NOVAGene compares modules based on the coordinated activity of their most influential genes.

For each module, we first identified its key hub genes using eigenvector centrality, computed via the networkX package (RRID: SCR_016864) in Python. Eigenvector centrality measures a gene’s influence within the network by considering its connections to other well-connected genes. This helps pinpoint genes that are not merely highly connected, but are central to the module’s overall organization and behavior. From these, we selected up to 10 of the most influential genes per module, including all available genes if fewer than ten were present, to focus on the core drivers of module activity.

To derive a robust and representative profile for each module’s core behavior, we performed Principal Component Analysis (PCA) on the expression profiles of these selected hub genes. PCA transforms the gene expression data into a set of linearly uncorrelated components, with the first principal component (PCA1) capturing the largest amount of variance. This PCA1 vector thus served as a signature reflecting the dominant collective activity and overall contribution of the module’s influential hubs. Using PCA1 helps to reduce noise and provide a single, summary measure of the most significant shared expression pattern among the module’s key regulatory genes.

To compare corresponding modules across different conditions (e.g., healthy vs. diseased), we computed Pearson’s correlation coefficients between their respective hub-derived PCA1 vectors. This direct comparison of these collective hub activity signatures allowed us to quantify both the magnitude and direction (positive or negative) of the association between module pairs. The resulting module correlation matrix provides a clear framework for understanding if modules align, diverge, or show inverse relationships in their core regulatory behavior between conditions. This approach moves beyond structural similarity to evaluate whether key regulatory or driver genes exhibit shared systems-level behavior, offering a complementary and mechanistically informative perspective on underlying phenotypic differences. A correlation of +1 indicates a perfect positive association in hub-gene activity, while a correlation of −1 reflects a complete inverse association. Statistical significance was determined at α=0.05 .

### 2.5 Benchmarking NOVAGene against baseline methods using synthetic datasets

To rigorously evaluate the performance of NOVAGene, we conducted a simulation study benchmarking our proposed method against three widely used gene co-expression analysis methods, namely: WGCNA (Langfelder and Horvath, 2008), DiffCoEx (Tesson et al., 2010), and DGCA (McKenzie et al., 2016). DiffCoEx and DGCA are recent alternative algorithms to WGCNA that employ different subsampling and nonlinear approach for gene co-expression analysis. This comparative study focused on the ability of each algorithm to accurately identify significant gene modules in datasets with varying variability or module overlap.

We constructed synthetic gene expression datasets consisting of 300 genes and 100 samples (50 healthy, 50 diseased), each with a well-defined modular structure. Specifically, 150 genes were simulated to co-express predominantly within the diseased group, and the remaining 150 within the healthy group. Gene expression values were drawn from a heavy-tailed log-normal distribution, incorporating nonlinear co-expression structures via cubic interactions. To emulate real-world biological variability, we introduced a tunable parameter p , which controlled the variance of gene expression across conditions. High values of p corresponded to increased variability and reduced module overlap, whereas low p values produced low-variability datasets with greater module overlap.

All four methods were applied to the synthetic gene expression datasets with low (p=0.1) and high (p=0.9) variability. Module detection results were evaluated against ground-truth assignments using precision and recall which are standard metrics in classification-based model evaluation. High precision indicates a low false positive rate, while high recall reflects strong sensitivity in recovering true differential co-expression signals. The R code used for data generation and method implementation is available in the Code Availability section to support full reproducibility of the analysis.

## 3 Results

### 3.1 Baseline clinical characteristics

A total of N=119 patients were included in this study, with N=39 patients classified as healthy and N=80 patients as diseased. Table 1 summarizes the clinical characteristics of these two groups. The mean age (p=0.750) and sex distribution (p=0.696) of the two groups were comparable, ensuring that differences observed in clinical characteristics could be attributed to disease status rather than demographic variability such as age and sex.

**TABLE 1:** Baseline clinical characteristics of healthy and diseased patients.

Variable	Healthy (N=39)	Diseased (N=80)	p	SMD
Age, mean (SD)	41.41 (10.86)	40.73 (11.16)	0.750	−0.062
Sex, n (% male)	17 (43.59)	39 (48.75)	0.696	−0.104
Mortality, n (%)	-	3 (3.75)	-	-
Duration of illness, mean (SD)	-	7.03 (10.83)	-	-
SOFA, mean (SD)	-	1.24 (1.69)	-	-

SOFA, sequential organ failure assessment; SMD, standardized mean difference.

The diseased group was further stratified into non-shock (N=61) and shock (N=19) outcomes. Table 2 presents a comparison of their demographic and baseline clinical characteristics. As expected, mortality (p=0.012) and multi-organ damage (p=0.005) were significantly higher in shock patients compared to non-shock. Notably, there is no significant difference in the duration of illness or hospitalization between the two groups (p=0.616) . Similar to the comparison between healthy and diseased patients, both the non-shock and shock groups demonstrated comparable age (p=0.462) and sex distribution (p=1.000) , minimizing the potential confounding effects of demographic variability.

**TABLE 2:** Baseline clinical characteristics of non-shock and shock patients.

Variable	Non-shock (N=61)	Shock (N=19)	p	SMD
Age, mean (SD)	40.23 (11.42)	42.32 (10.42)	0.462	0.191
Sex, n (% male)	30 (49.18)	9 (47.37)	1.000	0.036
Mortality, n (%)	0 (0.00)	3 (15.79)	0.012	0.612
Duration of illness, mean (SD)	6.79 (12.05)	7.79 (5.46)	0.616	0.107
SOFA, mean (SD)	0.89 (1.44)	2.37 (1.95)	0.005	0.866

SOFA, sequential organ failure assessment; SMD, standardized mean difference.

### 3.2 Comparing different methods of gene co-expression network analysis

**FIGURE 1:**
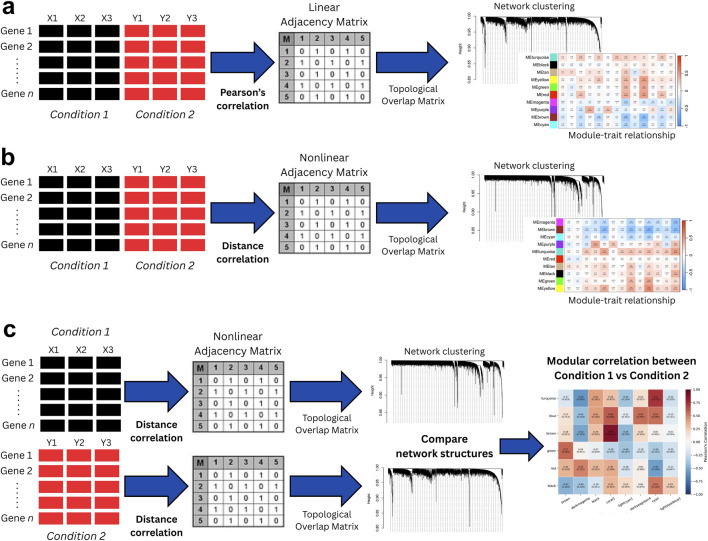
Comparison of gene co-expression network analysis methods: **(a)** Standard WGCNA, which constructs a network from pooled samples using a linear co-expression matrix; **(b)** Distance correlation-based approach, which also pools samples but captures nonlinear co-expression relationships; and **(c)** Our proposed method, which constructs separate networks for each condition based on nonlinear gene-gene interactions, enabling direct structural comparisons.

Figure 1 summarizes the workflows of different gene co-expression network analyses compared in this study. The standard WGCNA framework (Figure 1a) constructs a single co-expression network by aggregating all samples and applying a linear adjacency function to infer gene-gene relationships. A distance correlation-based approach (Figure 1b) similarly pools samples but replaces the linear adjacency function with a nonlinear distance correlation metric to capture more complex dependencies. In contrast, our proposed method (Figure 1c) builds independent gene co-expression networks for each condition, preserving distinct network architectures and enabling direct structural comparisons between biological states. We applied these methods to our local sepsis dataset, assessing their ability to detect significant biological modules that may underlie disease progression.

#### 3.2.1 Standard WGCNA pipeline

For baseline comparison, we constructed gene co-expression networks using Pearson’s correlation following the standard WGCNA pipeline. The gene expression dataset was first transformed into an adjacency matrix, capturing linear co-expression relationships across all samples (healthy vs. diseased, non-shock vs. shock). This resulted in a gene co-expression matrix containing pairwise gene correlations obtained from the combined samples of each comparison group. Hierarchical clustering was applied to the topological overlap matrix derived from the co-expression network to identify modules of highly interconnected genes, which are likely involved in coordinated biological processes.

**FIGURE 2:**
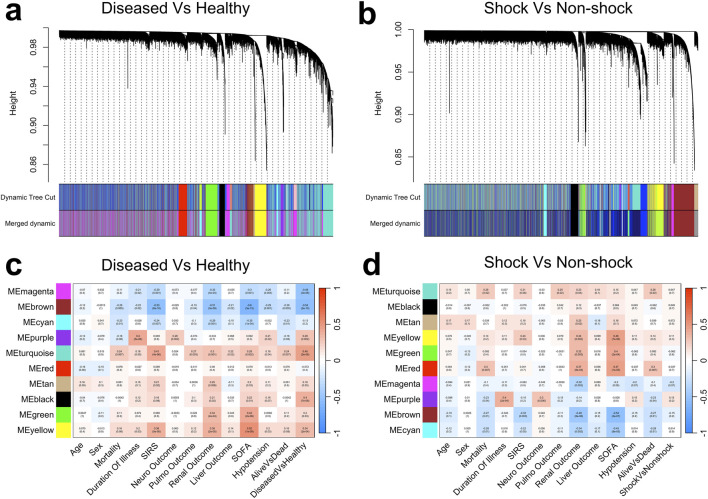
Cluster dendrograms depicting hierarchical clustering from the standard WGCNA for **(a)** diseased vs. healthy and **(b)** shock vs. non-shock groups. Correlation heatmaps illustrating the associations between identified gene modules and clinical traits for **(c)** diseased vs. healthy and **(d)** shock vs. non-shock groups, where color intensity reflects the strength and direction of correlations.

Figures 2a,b depict the hierarchical clustering of the linear gene co-expression matrix for the diseased vs. healthy and shock vs. non-shock comparison groups, respectively. To enhance statistical power, the standard WGCNA pipeline integrates all samples from the diseased and healthy states (including both shock and non-shock conditions); however, the resulting gene co-expression network does not distinguish clusters based on physiological state. Instead, the dendrogram reflects the inherent structure of gene co-expression patterns within the combined dataset.

To delineate disease-specific transcriptional signatures, gene clusters can be correlated with the desired clinical traits per sample. A positive correlation indicates upregulation in the diseased state relative to healthy controls, whereas a negative correlation suggests higher expression in the healthy condition. For instance, as shown in Figure 2c, the MEturquoise module exhibits the strongest positive association with disease state (R = 0.48, p<0.0001 ), whereas MEbrown demonstrates the most pronounced negative correlation (R = −0.52, p<0.0001 ). A similar pattern is observed for mortality, where MEturquoise is significantly enriched in individuals with fatal outcomes. In contrast, the gene modules identified in the shock vs. non-shock comparison (Figure 2d) exhibit weak correlations and do not reach statistical significance, suggesting a less distinct transcriptional signature in this grouping.

#### 3.2.2 Distance-correlation based WGCNA

A previous work of Hou et al. (2022) demonstrated that distance correlation captures complex, nonlinear relationships in gene co-expression networks beyond those detected by Pearson’s or Spearman’s correlation. To extend WGCNA’s conventional framework, we applied distance correlation to construct a nonlinear co-expression network from the same comparison groups. A similar hierarchical clustering was used to the topological overlap matrix derived from the nonlinear co-expression network to identify nonlinearly coexpressed gene modules.

**FIGURE 3:**
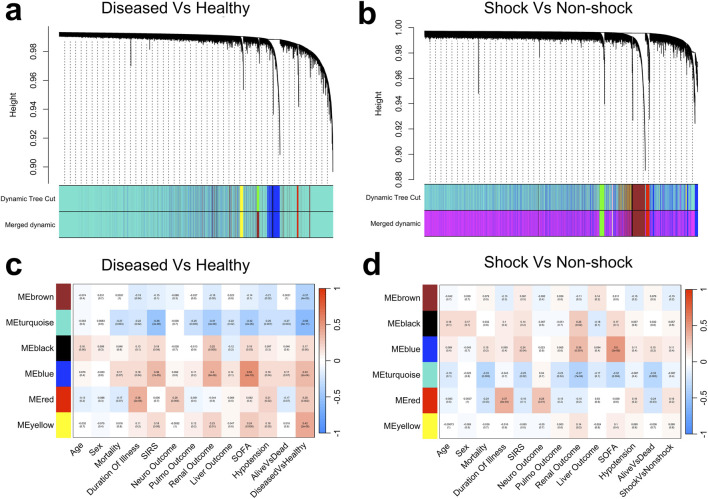
Cluster dendrograms depicting hierarchical clustering from the distance correlation-based WGCNA for **(a)** diseased vs. healthy and **(b)** shock vs. non-shock groups. Correlation heatmaps illustrating the associations between identified gene modules and clinical traits for **(c)** diseased vs. healthy and **(d)** shock vs. non-shock groups, where color intensity reflects the strength and direction of correlations.

Compared to the linear gene co-expression network, constructing the adjacency matrix using a normalized distance correlation (Equation 2) resulted in fewer gene modules in both the comparison groups (Figure 3). This reduction may stem from the fact that modules identified as distinct under linear assumptions may no longer be independent in a nonlinear framework, leading to their merging during hierarchical clustering.

However, similar to the conventional WGCNA approach, the nonlinear gene co-expression network consistently exhibited weak correlations and lacked statistical significance in the shock vs. non-shock comparison group. These findings suggest that even with a nonlinear co-expression framework, the standard WGCNA strategy of pooling samples within comparison groups (e.g. healthy vs. diseased or between disease subtypes) to increase statistical power may be insufficient for identifying significant gene modules in conditions with low heterogeneity and shared pathogenesis, such as sepsis and septic shock. The low heterogeneity between shock and non-shock groups is evident in their principal component analysis, where they exhibited an 87.21% overlap which is substantially higher than the divergence observed between diseased and healthy groups (Supplementary Figure S1).

#### 3.2.3 Modular comparison of nonlinear gene co-expression networks using NOVAGene

To address the limitations of conventional gene co-expression analysis in clinical datasets with shared pathogenesis such as sepsis and septic shock, we propose an alternative approach that constructs separate co-expression networks for each categorical state (e.g., healthy, diseased, non-shock, shock) rather than pooling the samples within comparison groups. Using distance correlation, we capture nonlinear gene-gene interactions that define biological processes within each state. The network was filtered by fitting a powerlaw distribution to enforce a heavy-tailed degree distribution, characteristic of biological networks (Supplementary Figure S2). Indeed, we showed in our goodness-of-fit tests that a truncated powerlaw distribution perfectly fits the distribution of our dataset among other heavy-tailed distributions such as log-normal and exponential (Supplementary Table S1). Gene modules were then identified through hierarchical clustering of the resulting nonlinear co-expression networks, as implemented in WGCNA.

**FIGURE 4:**
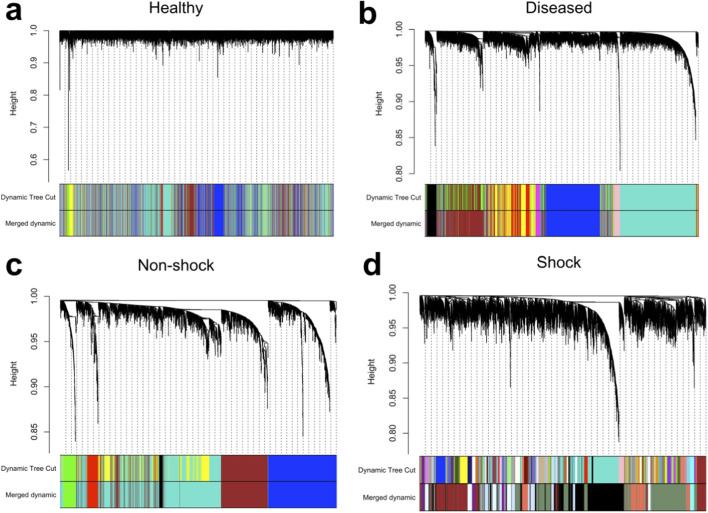
Cluster dendrograms depicting hierarchical clustering of the distance-correlated based gene co-expression matrix for individual conditions, namely: **(a)** healthy, **(b)** diseased, **(c)** non-shock sepsis, and **(d)** septic shock.

Figure 4 illustrates the hierarchical clustering of individual nonlinear co-expression networks. Notably, healthy samples exhibit weaker clustering compared to diseased states, suggesting lower gene-gene interconnectedness in the healthy condition. This finding may reflect a more tightly regulated transcriptional landscape in healthy individuals, whereas disease states exhibit greater co-expression and coordination among genes, potentially due to dysregulation or activation of specific pathways.d.

**FIGURE 5:**
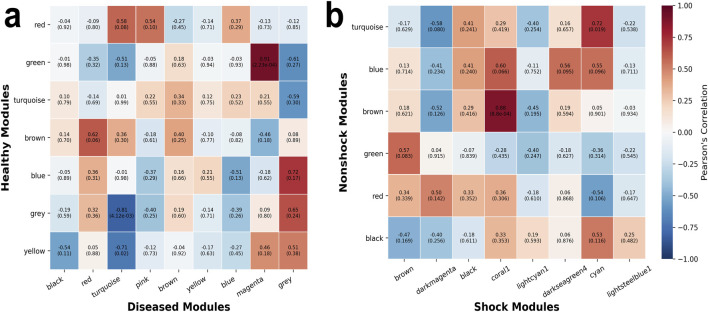
Modular correlation matrix of **(a)** diseased vs. healthy, and **(b)** shock vs. non-shock modules.

To compare modules within each group, NOVAGene employs an in-house module comparison algorithm that evaluates the eigenvector centrality of key hub genes (see Methods). A correlation value of +1 indicates a strong positive association, suggesting that the modules share coordinated gene expression patterns and may be involved in related or compensatory biological processes. Conversely, a correlation value of −1 reflects an inverse relationship, where the upregulation of one module is associated with the downregulation of the other, potentially indicating opposing regulatory mechanisms or distinct functional roles between states. Figure 5 illustrates the pairwise correlation analysis between diseased and healthy modules, as well as between shock and non-shock modules. For downstream functional analysis, we focused exclusively on module pairs exhibiting significant correlations (p<0.05) .

To identify enriched biological processes associated with these significant modules, we performed Gene Ontology (GO) enrichment analysis using DAVID (Huang et al., 2007). The results are summarized in Tables 3 and 4, detailing the key biological processes enriched within each comparison group.

**TABLE 3:** Gene Ontology (GO) Biological Process enrichment analysis for disease and healthy modules.

Modules	Pathway ID	GO BP terms	GeneRatio	p
**Diseased modules**				
Magenta	GO:0044772	Mitotic cell cycle phase transition	15/90	8.75E-06
	GO:0098813	Nuclear chromosome segregation	12/90	2.19E-05
	GO:0007059	Chromosome segregation	13/90	5.30E-05
	GO:0000280	Nuclear division	13/90	6.25E-05
	GO:0048285	Organelle fission	13/90	1.58E-04
**Turquoise**	GO:0007599	Hemostasis	66/1515	1.00E-16
	GO:0007596	Blood coagulation	63/1515	8.27E-16
	GO:0030168	Platelet activation	47/1515	8.27E-16
	GO:0050817	Coagulation	63/1515	1.55E-15
	GO:0042060	Wound healing	90/1515	6.28E-15
**Healthy modules**				
**Green**	GO:0015671	Oxygen transport	7/530	7.02E-08
	GO:0019755	One-carbon compound transport	11/530	9.08E-07
	GO:0015669	Gas transport	7/530	1.57E-06
	GO:0042744	Hydrogen peroxide catabolic process	8/530	1.64E-06
	GO:0051865	Protein autoubiquitination	10/530	1.89E-05
**Yellow**	GO:0030168	Platelet activation	23/812	5.54E-05
	GO:0007596	Blood coagulation	30/812	6.78E-05
	GO:0050817	Coagulation	30/812	6.78E-05
	GO:0042060	Wound healing	44/812	6.78E-05
	GO:0007599	Hemostasis	30/812	6.78E-05

**TABLE 4:** Gene Ontology (GO) Biological Process enrichment analysis for shock and non-shock modules.

Modules	Pathway ID	GO BP terms	GeneRatio	p
**Shock modules**				
**Cyan**	GO:0051607	Defense response to virus	22/86	6.90E-17
	GO:0140546	Defense response to symbiont	22/86	6.90E-17
	GO:0009615	Response to virus	23/86	8.00E-16
	GO:0140374	Antiviral innate immune response	7/86	7.48E-07
	GO:0045088	Regulation of innate immune response	14/86	2.15E-06
Coral1	GO:0019882	Antigen processing and presentation	16/475	1.48E-04
	GO:0031349	Positive regulation of defense response	32/475	1.29E-03
	GO:0019884	Antigen processing and presentation of exogenous antigen	9/475	4.48E-03
	GO:0002478	Antigen processing and presentation of exogenous peptide antigen	8/475	6.12E-03
	GO:0050867	Positive regulation of cell activation	26/475	7.79E-03
**Non-shock modules**				
**Brown**	GO:0002181	Cytoplasmic translation	40/579	3.39E-22
	GO:0030098	Lymphocyte differentiation	63/579	4.08E-22
	GO:1903131	Mononuclear cell differentiation	64/579	2.78E-20
	GO:0030217	T cell differentiation	50/579	2.31E-19
	GO:0046631	Alpha-beta T cell activation	35/579	1.59E-15
Turquoise	GO:0002274	Myeloid leukocyte activation	51/997	4.88E-14
	GO:0031349	Positive regulation of defense response	72/997	2.12E-12
	GO:0006909	Phagocytosis	46/997	2.08E-11
	GO:0002764	Immune response-regulating signaling pathway	70/997	9.22E-11
	GO:0045088	Regulation of innate immune response	62/997	3.29E-10

In the diseased vs. healthy comparison, modular correlations reveal significant biological relationships as shown in Figure 5. The positive correlation between the diseased module (Magenta: nuclear division) and the healthy module (Green: oxygen transport) suggests that cell cycle dysregulation in disease may be linked to altered oxygen transport, possibly as a compensatory response to increased metabolic demands or hypoxic conditions. Interestingly, the negative correlation between two hemostasis-related modules, namely Turquoise (hemostasis, blood coagulation) in diseased and Yellow (hemostasis, blood coagulation) in healthy – suggests that while coagulation is an active process in both states, their regulation differs fundamentally, with disease-associated coagulation possibly reflecting pathological thrombosis rather than physiological hemostasis (Williams et al., 2024; Cao et al., 2022).

We observed significant positive correlations in comparing the two outcomes (i.e. septic shock and non-shock groups) of the diseased patients (Figure 5). The correlated modules are predominantly associated with immune responses, highlighting shared immunological processes between the two groups (Table 4). The correlation between Coral1 (shock, antigen processing and presentation) and Brown (non-shock, T cell differentiation, immune signaling pathways) indicates that antigen presentation and T cell differentiation are functionally linked across groups. Similarly, the positive correlation between Cyan (shock, viral response and defense response to virus) and Turquoise (non-shock, myeloid activation and phagocytosis) suggests that innate immune activation, particularly involving phagocytosis and antiviral defense, is a shared feature in both groups. Modules enriched in non-shock patients, including cytoplasmic translation, lymphocyte differentiation, and myeloid activation, likely represent early immune activation. In contrast, shock-associated modules, characterized by antiviral responses, antigen presentation, and upregulation of defense pathways, reflect later-stage adaptive immunity involving clonal expansion of CD8^+^ T cells and B cells. Notably, the absence of negatively correlated modules implies that the transcriptomic profiles resulting to these two clinical outcomes may not represent distinct or opposing pathophysiological mechanisms, but rather reflect subtle yet different manifestations along a common disease continuum (Meyer and Prescott, 2024; Jacobi, 2021).

### 3.3 Benchmarking NOVAGene using synthetic datasets

To evaluate the effectiveness of NOVAGene in identifying condition-specific gene modules, we applied the method to synthetic gene expression datasets designed with varying levels of variability. We showed the summary of the precision and recall metrics in Table 5.

**TABLE 5:** Performance of NOVAGene and baseline methods on synthetic gene expression datasets with low and high variability.

Methods	Low variability (p=0.1)		High variability (p=0.9)	
	Precision (%)	Recall (%)	Precision (%)	Recall (%)
WGCNA	NA	0.00	100.00	100.00
DiffCoEx	NA	0.00	96.64	96.00
DGCA	0.00	0.00	55.22	49.33
**NOVAGene**	**38.56**	**71.43**	**49.32**	**85.85**

The bolded values correspond to the performance metrics of NOVAGene.

In the low-variability setting (p=0.1) , where traditional co-expression methods often underperform, NOVAGene was uniquely able to identify relevant gene modules associated with the diseased and healthy conditions. Specifically, it detected a total of ten modules across both networks, achieving a precision of 38.56% and a recall of 71.43%. In contrast, both WGCNA and DiffCoEx failed to identify statistically significant modules (p>0.05) , and DGCA, although detecting a “brown” module, misassigned it to the incorrect phenotype (Supplementary Figure S4).

In the high-variability setting (p=0.9) , WGCNA and DiffCoEx achieved near-perfect performance, with both precision and recall exceeding 96%, as expected in datasets with clearly separated co-expression patterns. Notably, NOVAGene maintained high recall (85.85%) in this setting, surpassing DGCA, which achieved only 49.33% recall. Although NOVAGene’s precision (49.32%) was lower than that of WGCNA and DiffCoEx, its strong sensitivity suggests that it remains effective in detecting true signal even in more heterogeneous or high variability conditions.

The results of this simulation study are consistent with those observed in our local sepsis dataset. This therefore highlights the robustness and practical utility of NOVAGene in uncovering biologically meaningful gene modules, especially in complex or subtle expression landscapes where traditional methods may fail.

## 4 Discussion

WGCNA has become a cornerstone in gene co-expression network analysis, offering a computationally efficient and scalable framework for large-scale transcriptomic and genomic studies. Its reliance on linear approximations makes it widely accessible; however, gene interactions in biological systems are often inherently nonlinear, potentially limiting the ability of linear models to fully capture complex regulatory dynamics (Hou et al., 2022; Zhang and Wong, 2022). With advances in high-performance computing enabling large-scale computations at unprecedented speeds, revisiting these assumptions is both timely and necessary. While incorporating nonlinear dynamics into network analysis may come with greater computational demands, it holds the potential to uncover deeper insights into the complex regulatory mechanisms underlying disease pathophysiology.

We utilized a local sepsis dataset to assess the efficacy of WGCNA in examining gene expression across samples exhibiting varying degrees of biological variability (Supplementary Figure S1). Specifically, we compared gene expression profiles between diseased and healthy individuals, representing a high-variability dataset characterized by minimal overlap (11.02%) in PCA plots. Conversely, the comparison between shock and non-shock groups, both within a disease spectrum, exhibited low variability, as evidenced by substantial overlap in PCA (87.21%) , reflecting their shared pathophysiology.

Our findings indicate that WGCNA effectively identifies significant co-expression modules in high-variability datasets, aligning with its established utility in capturing coordinated gene expression changes across diverse conditions (Stanford et al., 2020; Saelens et al., 2018). However, in low-variability contexts, such as the shock versus non-shock comparison, its reliance on linear correlations may limit its sensitivity to subtle gene expression differences, resulting in reduced efficacy of detecting nuanced gene interactions within datasets exhibiting minimal variability (Hou et al., 2022; 2021).

To address this challenge, we employed distance correlation, a statistical measure that captures both linear and nonlinear dependencies between variables, to construct an alternative adjacency matrix as input for the standard WGCNA pipeline (Hou et al., 2022). Unlike Pearson correlation, which only detects linear relationships, distance correlation assesses the degree of association between variables by measuring how their pairwise distances co-vary in a high-dimensional space. This allows it to uncover complex, nonlinear gene-gene interactions that traditional correlation measures may overlook.

By integrating distance correlation into WGCNA, we aimed to better preserve nonlinear relationships within the co-expression network. However, despite this modification, our results showed that WGCNA still failed to identify significant co-expression modules differentiating shock from non-shock samples (Figure 3D). We attribute this limitation to WGCNA’s intrinsic approach of pooling all samples to construct a single network, which may dilute nonlinear network signatures when applied to datasets where the underlying network structures are highly similar. This suggests that conventional WGCNA, even when supplemented with nonlinear correlation measures, may not fully capture the subtle transcriptional shifts that distinguish progressive disease states within the same pathophysiological continuum.

Thus, we proposed a novel approach called NOVAGene (or Nonlinear Overlapping Variability Analysis of Gene Networks) for analyzing nonlinear gene co-expression networks which overcomes the limitations of standard WGCNA. Instead of pooling samples into a single network, we constructed and analyzed individual nonlinear gene networks for each condition. We then compared their structural differences by identifying key hub genes, module compositions, and enriched biological processes. Unlike the conventional WGCNA pipeline, which struggles to differentiate gene expression patterns in low-variability datasets, NOVAGene effectively discriminates both high- and low-variability samples.

While NOVAGene consistently identifies the same enriched biological processes as in the standard WGCNA and distance correlation-based WGCNA, such as hemostasis and oxygen transport (Supplementary Tables S2 and S3), it more notably provides new insights into the immune dynamics distinguishing the transcriptome of those diseased patients who progressed to shock from those who remained non-shock. Our analysis reveals that while non-shock and shock outcomes share core immune processes, they differ in regulatory dynamics particularly in terms of temporal patterns of immune activation.

In COVID-19, early immune responses can still play a protective role in viral clearance. However, when these responses become exaggerated and dysregulated, often resulting in a cytokine storm, they contribute to the worsening of disease outcomes (Qin et al., 2022). The positive correlation between the non-shock and shock modules may reflect significant propensity to heightened innate immune activation from presistent antigen activation (Liu et al., 2022; Delano and Ward, 2016). The transition from a controlled to a dysregulated immune response in sepsis shows its impact on both innate and adaptive immunity, often culminating in sustained immunosuppression (Delano and Ward, 2016). Intramodular analysis remains necessary to further investigate the distinct immune components that drive the different pathways involved in non-shock and shock outcomes.

While sepsis is primarily characterized by immune dysregulation, it also involves other systemic processes such as endothelial dysfunction, coagulation abnormalities, and mitochondrial dysfunction (Arora et al., 2023; Nedel et al., 2023). All of these factors contribute to the complexity of sepsis. Thus, relying solely on PBMCs for transcriptomic analysis may provide limited interpretation and an incomplete picture on the pathogenesis of sepsis. We recommend therefore to use transcriptomic data from other cell types such as endothelial cells to enhance biomarker discovery and improve prognostication in sepsis.

The results of our simulation study also further underscores the robustness and practical utility of NOVAGene, particularly in identifying biologically relevant gene modules in low-variability or complex datasets where traditional methods are less effective. Although NOVAGene exhibited a higher false positive rate compared to other methods, this trade-off is acceptable at this discovery stage since the identified modules can subsequently be validated against known biological pathways in downstream analyses. More importantly, NOVAGene offers an interpretable and biologically plausible set of gene networks potentially associated with disease phenotypes with low heterogeneity such as in sepsis and septic shock.

Despite its advantages, NOVAGene is not without limitations. First, the computational requirement is greater than that of standard WGCNA, as it involves additional steps for network comparison rather than a direct association with clinical traits. Second, the absence of sample pooling may reduce statistical power compared to standard WGCNA. The minimum sample size required for robustness remains to be fully explored; however, even with as few as 15 shock samples, we identified biologically meaningful modules consistent with previous literature. We anticipate that increasing the sample size will further enhance the resolution and sensitivity of NOVAGene, allowing for the identification of additional and more robust functional modules.

Nonetheless, NOVAGene’s multistep preprocessing pipeline, designed to enhance data quality and network robustness, yields biologically plausible network structures that are more resilient to random noise and local fluctuations. This ensures that the detected co-expression patterns reflect true underlying biological signals rather than spurious correlations, even in smaller or noisier datasets.

## 5 Conclusion

This study presents a novel approach to gene co-expression network analysis called NOVAGene for identifying significant biological pathways across both high- and low-variability transcriptomic datasets. By enhancing gene co-expression analysis of a local sepsis dataset, we offered a more refined understanding of the pathogenesis of sepsis and septic shock – conditions often characterized by limited transcriptional variability. These insights highlight the utility of such framework in improving the prognostication of sepsis outcomes, thereby enabling timely and targeted immunomodulatory interventions.

## Data Availability

The R and Python codes used in this study are available in the following GitHub repository: https://github.com/jcbacong/NOVAGene.
