# Supplementary material for: NOVAGene: a nonlinear approach to improve biological resolution in weighted gene co-expression networks for low-variability datasets
Source: Front Bioinform. 2026 May 12;6:1813626. doi: 10.3389/fbinf.2026.1813626 (PMC13201898; doi:10.3389/fbinf.2026.1813626)
Supplement: Supplementary file 1 [file DataSheet1.pdf]

Supplementary Material

1 CODE AVAILABILITY

The source code (R and Python) used for data generation and methodological implementation in this study are openly accessible via the following GitHub repository: https://github.com/jcbacong/NOVAGene.

2 SAMPLE VARIABILITY WITHIN COMPARISON GROUPS

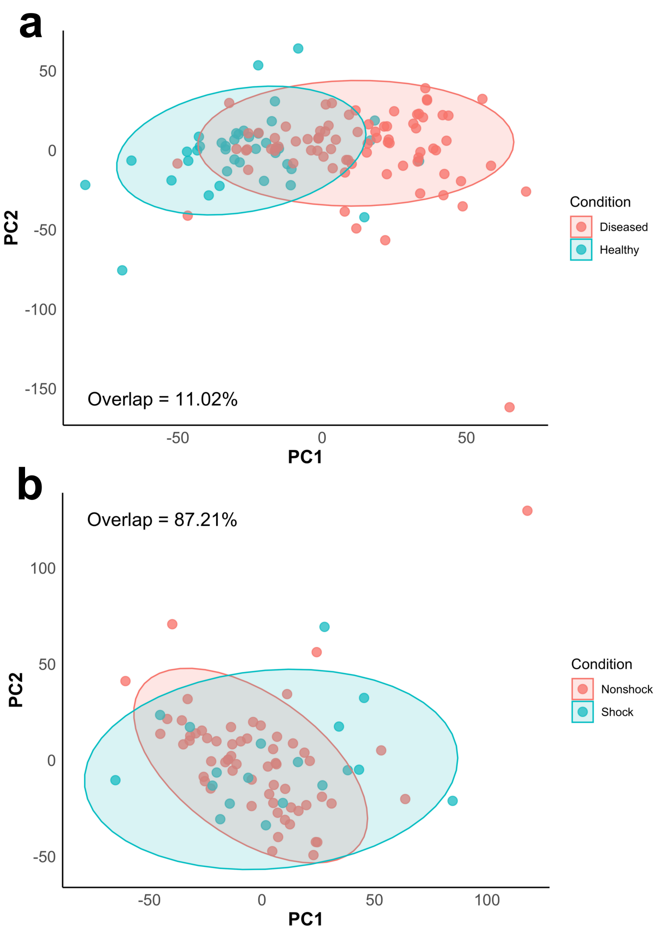

Figure S1. Principal component analysis (PCA) of the comparison groups: (a) diseased vs. healthy and (b) shock vs. non-shock. The shock vs. non-shock group exhibits a high degree of overlap (87.21%), whereas the diseased vs. healthy group shows minimal overlap (11.02%), indicating greater similarity or low heterogeneity in the gene expression between shock and non-shock conditions.

3 NETWORK CONSTRUCTION USING A TRUNCATED POWERLAW DISTRIBUTION

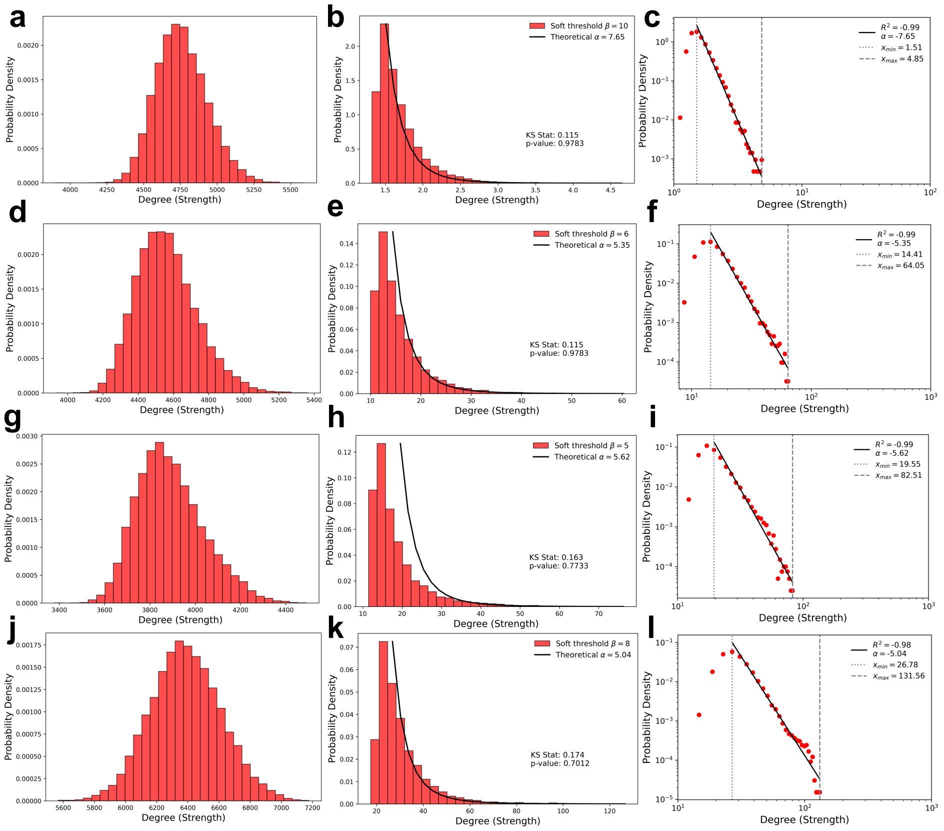

Figure S2. Network filtering of raw distance correlation values for (a–c) healthy, (d–f) diseased, (g–i) non-shock, and (j–l) shock samples. A soft-thresholding function in WGCNA was used to transform the initial distribution into a heavy-tailed distribution using the exponent β, emphasizing high-degree connections. A power-law distribution was then fitted with theoretical parameters α and $\mathrm{x_{min}}$. Nodes with degrees below $\mathrm{x_{min}}$ were pruned to improve signal-to-noise ratio while retaining a powerlaw behavior that is a characteristic feature of many biological networks.

4 NETWORK PARAMATERS AND GOODNESS-OF-FIT TEST

In all four networks, the truncated power-law distribution passed the Kolmogorov–Smirnov (KS) statistical test ($p > 0.05$), supporting its adequacy. Moreover, we have also computed the log-likelihood ratio (LLR) comparing the truncated power-law (tPL) fit to other alternative heavy-tailed distributions such as log-normal (LN) and exponential (Exp) distributions. The results are summarized below.

Table S1. Summary of fitted parameters and comparative model statistics from network construction using a truncated power-law distribution.

Network	α	x_{min}	KS (p-value)	LLR (tPL vs LN)	LLR (tPL vs Exp)
Healthy	-7.65	1.51	0.115 (p = 0.978)	-1.005 (p = 0.404)	40.093 (p = 0.001)
Diseased	-5.35	14.41	0.115 (p = 0.978)	-1.459 (p = 0.271)	18.040 (p = 0.025)
Nonshock	-5.62	19.55	0.163 (p = 0.773)	-1.005 (p = 0.404)	40.093 (p = 0.001)
Shock	-5.04	26.78	-0.472 (p = 0.519)	17.532 (p = 0.519)	17.532 (p = 0.013)

We showed that the truncated power-law distribution consistently fits the data better than the exponential model across all networks. Although the log-normal distribution provided comparable fits (non-significant LLRs), it did not significantly outperform the power-law model. Therefore, the assumption of a truncated power-law distribution for filtering appears appropriate and justified.

5 CONSTRUCTION OF SYNTHETIC DATASETS WITH TUNABLE VARIABILITY

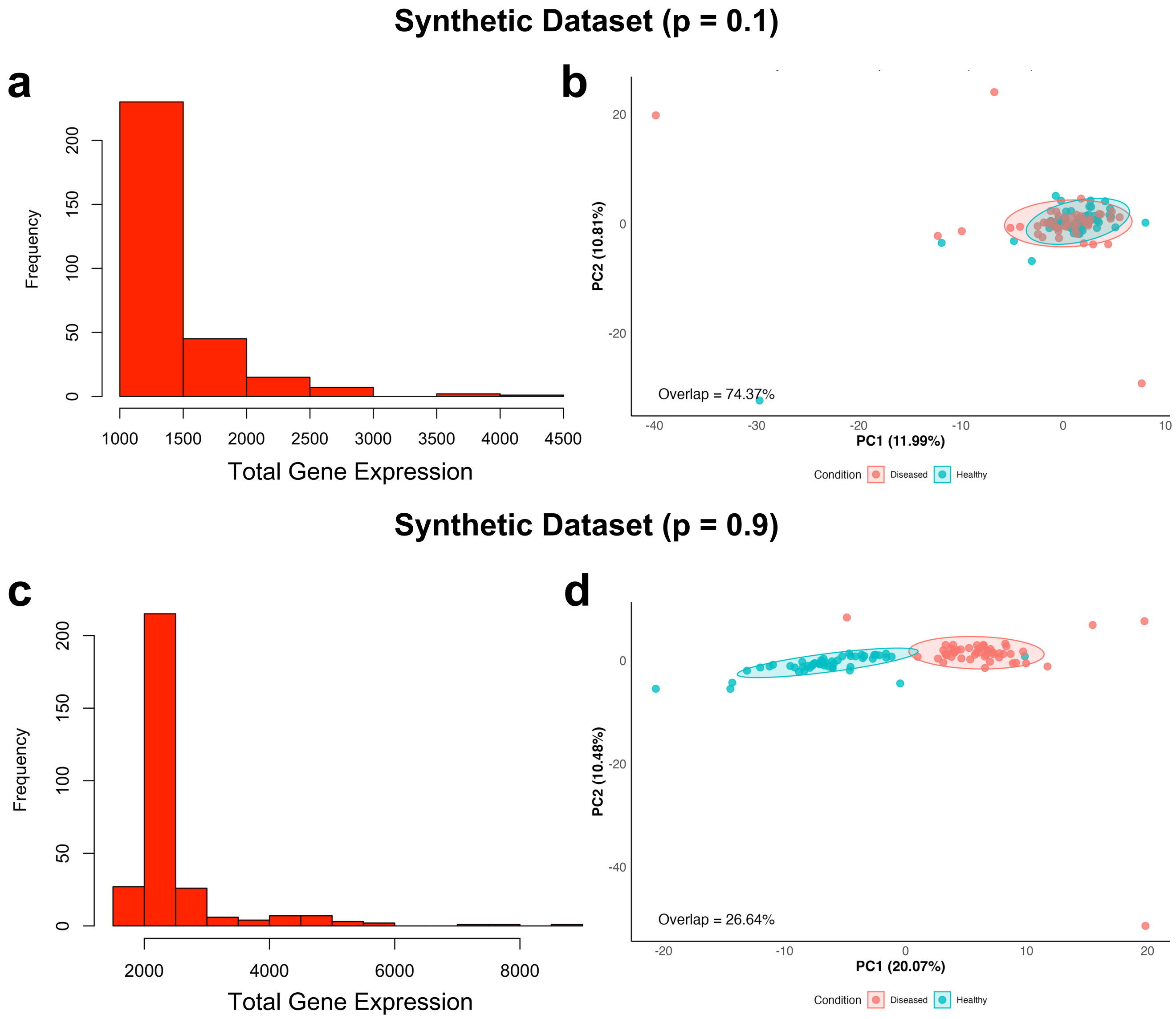

Figure S3. The synthetic datasets consist of 300 genes and 100 samples (50 healthy, 50 diseased) with a predefined modular architecture. Gene expression values were sampled from a heavy-tailed log-normal distribution and incorporate nonlinear co-expression via cubic interaction terms. A tunable parameter p was introduced to achieve real-world biological variability, where (a-b) $p = 0.1$ shows low variability with 74.37% overlap and (c-d) $p = 0.9$ produces high variability with 26.64% overlap.

6 MODULE DETECTION OF BASELINE METHODS ON SYNTHETIC GENE EXPRESSION DATASETS

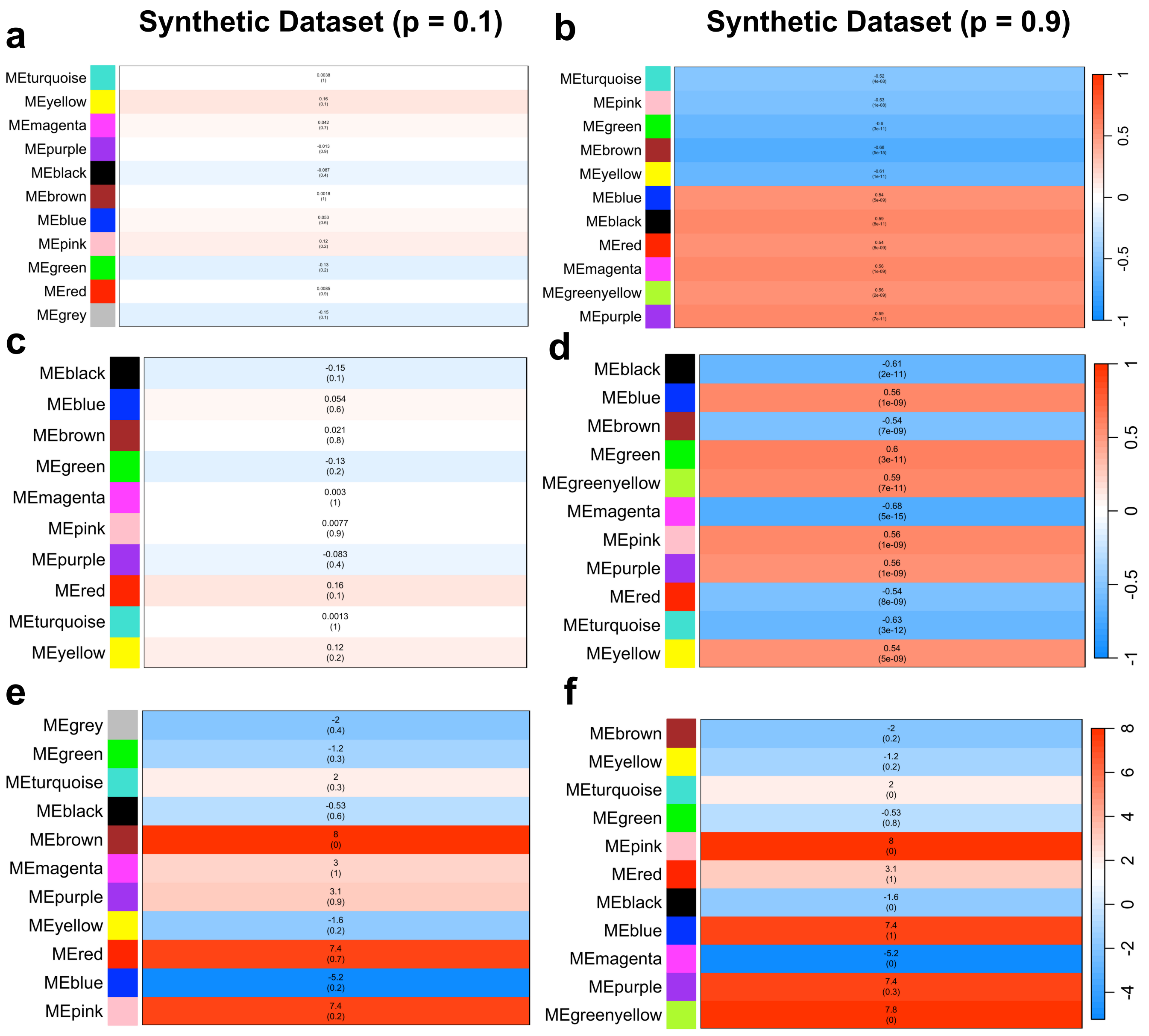

Figure S4. A simulation study was conducted to benchmark the performance of NOVAGene against three baseline methods, namely: (a-b) WGCNA, (c-d) DiffCoEx, and (e-f) DGCA, on synthetic gene expression datasets with low ($p = 0.1$) and high ($p = 0.9$) variability. In the low-variability condition, both WGCNA and DiffCoEx failed to identify statistically significant modules ($p > 0.05$), while DGCA detected a "brown" module but incorrectly assigned it to the non-diseased phenotype.

7 MODULE ANALYSIS OF NOVAGENE ON SYNTHETIC GENE EXPRESSION DATASETS

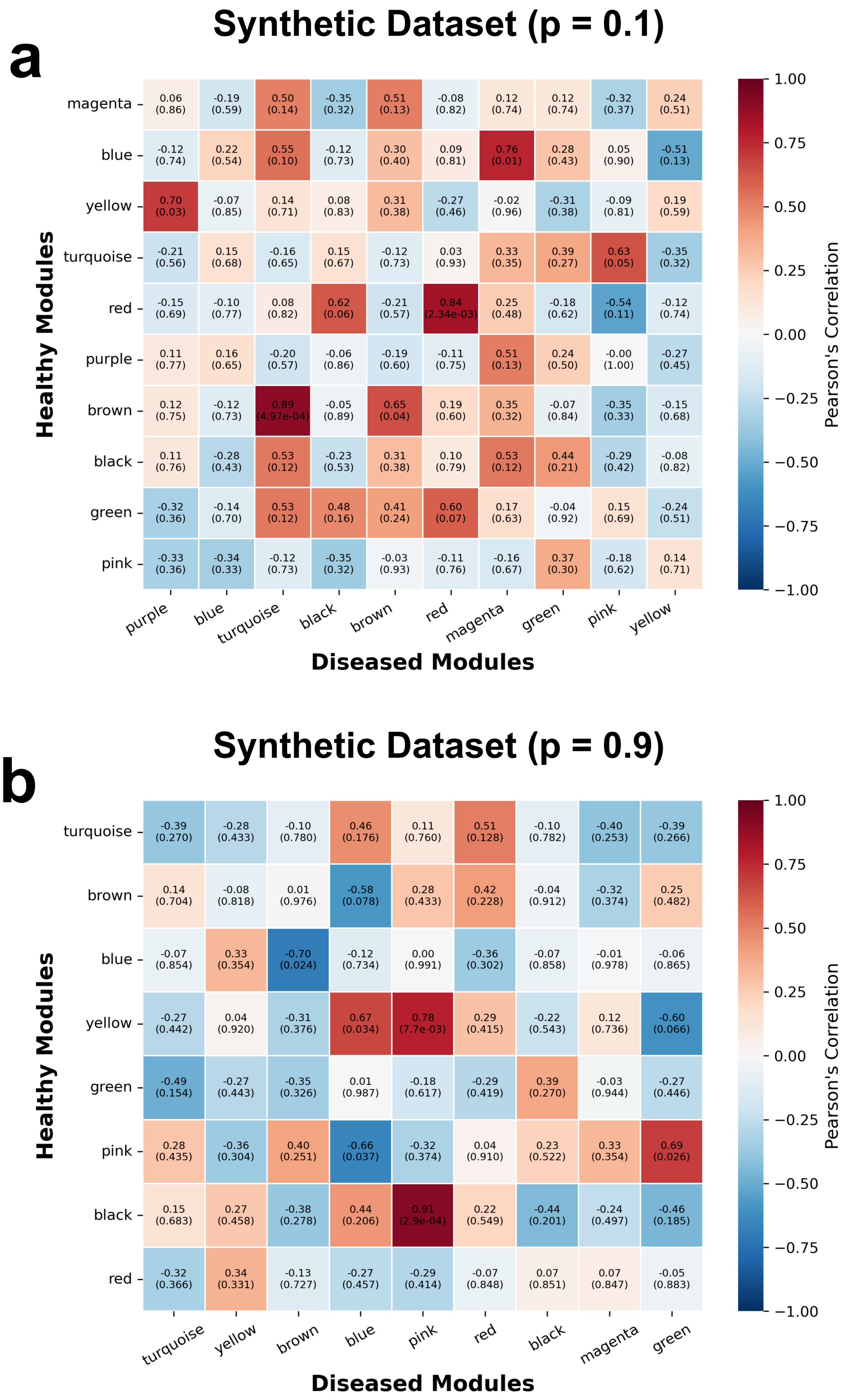

Figure S5. NOVAGene successfully identified relevant gene modules associated with the diseased condition in both (a) low-variability ($p = 0.1$) and (b) high-variability ($p = 0.9$) settings. Under low variability settings, where baseline methods underperformed, NOVAGene detected 10 disease-associated modules. In the high-variability condition, it identified 8 modules, demonstrating consistent performance across different levels of expression variability.

8 FUNCTIONAL ANALYSIS OF STANDARD WGCNA MODULES

Table S2. Gene Ontology (GO) Biological Process (BP) Terms for Diseased vs Healthy Comparison Group

Modules	Pathway ID	GO BP Terms	GeneRatio	p-value
**Positively Correlated Modules**				
**Turquoise**	GO:0022407	Regulation of cell-cell adhesion	216/5281	1.27E-10
	GO:0007159	Leukocyte cell-cell adhesion	188/5281	1.66E-10
	GO:0045785	Positive regulation of cell adhesion	210/5281	1.66E-10
	GO:0002768	Immune response-regulating cell surface receptor signaling pathway	161/5281	1.66E-10
	GO:0002696	Positive regulation of leukocyte activation	173/5281	1.82E-10
**Black**	GO:0007059	Chromosome segregation	51/300	1.34E-26
	GO:0000280	Nuclear division	51/300	4.50E-26
	GO:0098813	Nuclear chromosome segregation	43/300	7.55E-25
	GO:0000070	Mitotic sister chromatid segregation	35/300	7.95E-25
	GO:0048285	Organelle fission	51/300	2.28E-24
**Yellow**	GO:0042060	Wound healing	67/650	4.75E-26
	GO:0007599	Hemostasis	48/650	3.75E-24
	GO:0007596	Blood coagulation	47/650	7.30E-24
	GO:0030168	Platelet activation	37/650	1.35E-23
	GO:0050817	Coagulation	47/650	1.98E-23
**Negatively Correlated Modules**				
**Brown**	GO:0007608	Sensory perception of smell	115/2997	1.40E-07
	GO:0050911	Detection of chemical stimulus involved in sensory perception of smell	106/2997	1.53E-06
	GO:0050907	Detection of chemical stimulus involved in sensory perception	115/2997	1.99E-06
	GO:0045229	External encapsulating structure organization	83/2997	3.40E-06
	GO:0030198	Extracellular matrix organization	82/2997	4.96E-06
**Magenta**	GO:0032543	Mitochondrial translation	52/3493	8.63E-09
	GO:0042773	ATP synthesis coupled electron transport	43/3493	2.66E-08
	GO:0042775	Mitochondrial ATP synthesis coupled electron transport	43/3493	2.66E-08
	GO:0019646	Aerobic electron transport chain	40/3493	5.90E-08
	GO:0140053	Mitochondrial gene expression	58/3493	1.71E-07

9 FUNCTIONAL ANALYSIS OF DISTANCE CORRELATION-BASED WGCNA MODULES

Table S3. Gene Ontology (GO) Biological Process (BP) terms associated with positively and negatively correlated modules.

Modules	Pathway ID	GO BP Terms	GeneRatio	p-value
**Positively Correlated Modules**				
**Blue**	GO:0042060	wound healing	80/1155	3.93E-16
	GO:0007596	blood coagulation	53/1155	1.77E-14
	GO:0030168	platelet activation	40/1155	1.77E-14
	GO:0050817	coagulation	53/1155	2.55E-14
	GO:0007599	hemostasis	53/1155	3.05E-14
**Yellow**	GO:0007059	chromosome segregation	37/168	3.20E-26
	GO:0098813	nuclear chromosome segregation	31/168	1.07E-23
	GO:0000280	nuclear division	35/168	2.02E-23
	GO:0048285	organelle fission	35/168	5.76E-22
	GO:0000819	sister chromatid segregation	25/168	2.01E-20
**Red**	GO:0051607	defense response to virus	41/113	3.85E-40
	GO:0140546	defense response to symbiont	41/113	3.85E-40
	GO:0009615	response to virus	42/113	5.83E-37
	GO:0045088	regulation of innate immune response	27/113	8.28E-18
	GO:0048525	negative regulation of viral process	14/113	1.05E-13
**Negatively Correlated Modules**				
**Turquoise**	GO:0007601	visual perception	196/11853	2.29E-19
	GO:0050953	sensory perception of light stimulus	199/11853	2.73E-19
	GO:0048568	embryonic organ development	370/11853	6.86E-19
	GO:0023061	signal release	387/11853	1.05E-16
	GO:0048732	gland development	357/11853	1.72E-16
**Brown**	GO:0032543	mitochondrial translation	28/897	5.99E-08
	GO:0140053	mitochondrial gene expression	31/897	7.36E-08
	GO:0009060	aerobic respiration	32/897	1.39E-06
	GO:0015980	energy derivation by oxidation of organic compounds	44/897	1.39E-06
	GO:0045333	cellular respiration	35/897	4.35E-06
